# The complete chloroplast genome sequence of *Brainea insignis* (Blechnaceae)

**DOI:** 10.1080/23802359.2020.1756959

**Published:** 2020-05-12

**Authors:** Jiaojun Yu, Hongyu Wang, Hongjin Dong, Yuanping Fang, Jun Xiang

**Affiliations:** a Hubei Key Laboratory of Economic Forest Germplasm Improvement and Resources Comprehensive Utilization, Huanggang Normal University, Huanggang, China; b Hubei Collaborative Innovation Center for the Characteristic Resources Exploitation of Dabie Mountains, Huanggang, China

**Keywords:** *Brainea*, complete chloroplast genome, Blechnaceae, phylogeny

## Abstract

*Brainea insignis* (Hooker) J. Smith, a member of Blechnaceae, is a rare and endangered species in tropical Asia. Here we assembled and annotated the complete chloroplast (cp) genome. It is 149,730 bp in length and encodes 88 protein-coding genes, 36 transfer RNA (tRNA) genes and eight ribosomal RNA (rRNA) genes. This chloroplast genome sequencing offers a useful resource for future conservation genetics and phylogenetic studies.

*Brainea* is a monotypic genus endemic to tropical regions of Asia. The only species *B. insignis* is widely distributed in the damp and exposed hillsides at altitudes of 300–1700 m of south tropical China and other tropical Asian countries (Wang et al. 2013).

This taxon is listed as endangered and rare protected species in the Chinese Dangerous Plant Red Paper (category II) (Fu 1992). In present study, the completed chloroplast genome sequence of *B. insignis* is reported contributing for better understanding its evolution and population genetics, and also providing significant information for the phylogeny of Ferns.

Genomic DNA was extracted from fresh leaves of a seedling of *B. insignis* from Simao, Puer, Yunnan, China (100°58′45.80″E, 22°45′0.50″N, 1163 m; *Zhao qianru* et al.*19076*, 2019-2-7; KUN), the total genomic DNA was isolated according to a modified CTAB method (Doyle and Doyle 1987). Those leaves were stored in the refrigerator at −80 °C. Total genome DNA of *B. insignis* was sequenced by Illumina Hiseq 2500 Sequencing System (Illumina, Hayward, CA) to construct the shotgun library and assembled through the NOVOPlasty software (Dierckxsens et al. 2017). The low quality sequences were filtered out Using CLC Genomics Workbench v8.0 (CLC Bio, Aarhus, Denmark) and then reconstructed the chloroplast genome by using MITObim v1.8 (University of Oslo, Oslo, Norway; Kaiseraugst, Switzerland) (Hahn et al. 2013). The complete chloroplast genome of *B. insignis* was annotated in Geneious ver. 10.1 (http://www.geneious.com, Kearse et al. 2012) and then submitted to GenBank (accession no. MT265386). The genome annotation was performed by aligning with the cp genomes of relatively related species.

The size of chloroplast genome of *B. insignis* is 149,730 bp, including a large single-copy (LSC) region of 81,445 bp and a small single-copy (SSC) region of 21,509 bp separated by a pair identical inverted repeat regions (IRs) of 23,388 bp each. A total of 132 genes were successfully annotated containing 88 protein-coding genes, 36 tRNA genes and 8 rRNA genes. GC content of the whole genome, IRs, LSC and SSC regions are 41.5%, 44.8%, 40.5% and 38.0%, respectively. GC content of IRs region is the highest. 21 genes contain one intron, while 4 genes have two introns. The complete chloroplast genome sequence of *B. insignis* and other species from Blechnaceae were used to construct phylogenetic tree (Figure 1). The sequences were initially aligned using MAFFT (Katoh and Standley 2013) and then visualized and manually adjusted using BioEdit (Hall 1999). Take the plastome of *Equisetum arvense* (GenBank: GU191334) as an out-group, a maximum likelihood analysis was performed with RAxML version 8 program (Alexandros 2014) using 1000 bootstrap. The result shows the position of *B. insignis* in the ferns, which is consistent with previous molecular results (Lu et al. 2015; Wei et al. 2017).

**Figure 1.**
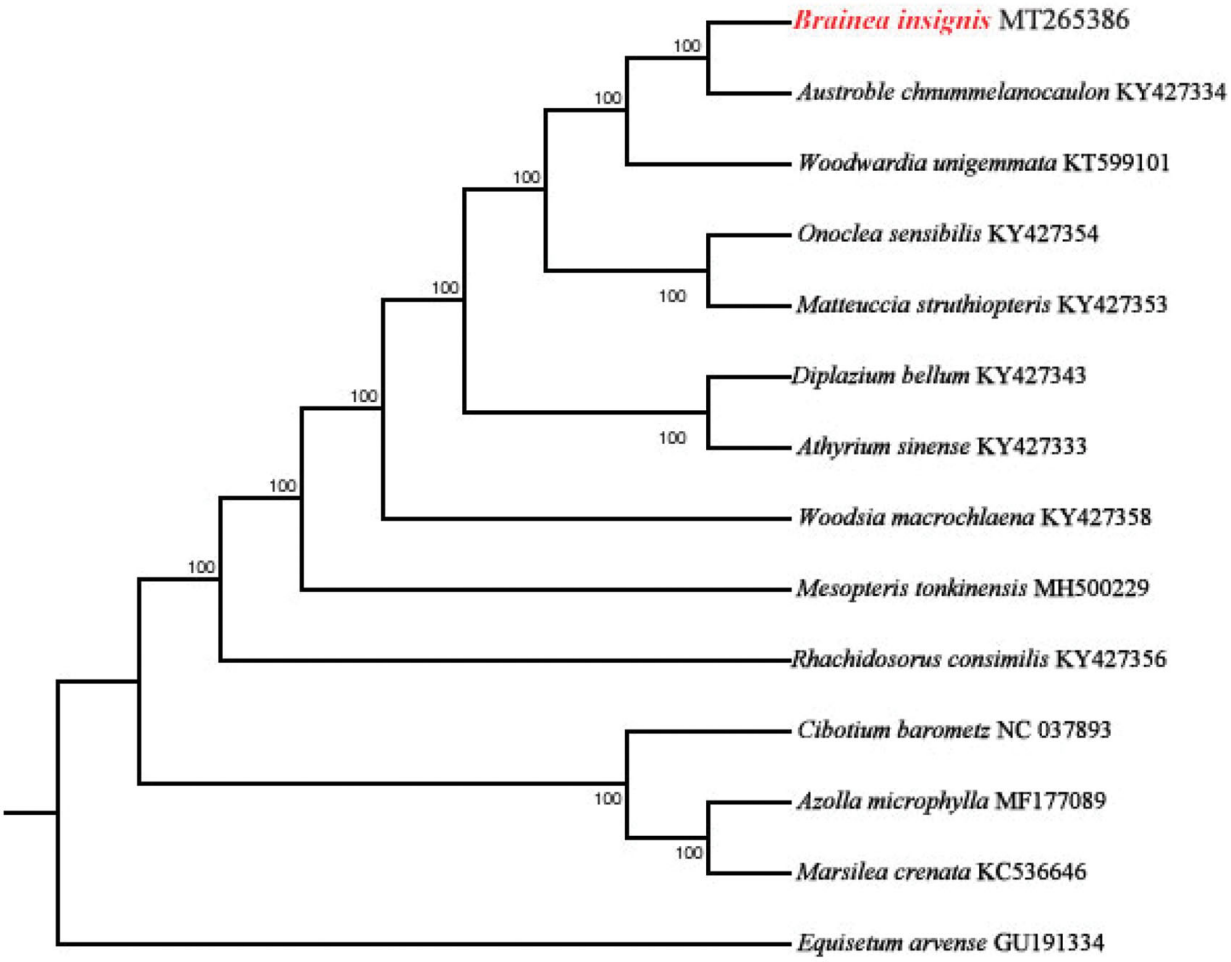
Maximum likelihood phylogenetic tree for *Brainea insignis* based on 13 other ferns species complete chloroplast genomes. The number on each node indicates bootstrap support value.

## Data Availability

The data that newly obtained at this study are available in the NCBI under accession number of MT265386.
